# Hotel Dieu

**Published:** 1829-01-01

**Authors:** 


### Periscope;

OR,

### CIRCUMSPECTIVE REVIEW.

THE

### HOSPITAL REPORTER,

OR,

### CLINICAL REVIEW.

No. I.

" Ore trahit quodcunque potest, atque addit acervo."

### I.

### HOTEL DIEU.

#### I. On Traumatic Gangrene.

To Baron Larrey we owe the division of gangrene into constitutional and traumatic, a division important alike in theory and practice. Though it is not our intention, nor indeed in our power, in the limited space of a clinical article, to enter on a laboured or learned disquisition, yet, before we proceed to particular cases, it is absolutely necessary to indulge in a few, and but few, observations.

It had been found, by experience, that the removal, by art, of a mortified, or rather, a mortifying limb, tended to hasten the fatal event. This was the general result of experience, and from it was deduced as a general rule—to defer amputation till the system itself seemed to call for it, by forming the line of separation, and, as it were, chalking out a path for the surgeon. Founded in truth, the principle, if carried to its utmost extent, is evidently fraught with error, because it supposes that gangrene is ever a constitutional affection, and never a local one. We can easily conceive, that the mortification which creeps upon the limb of an octogenarian, commencing at the toe, and slowly but steadily mounting upwards, depends upon a want of power in the constitution at large, and is not to by cured by lopping off a part. This, as we said before, we can easily conceive; the vis vitæ is enfeebled, by age, or other debilitating causes; the arteries are probably ossified; the heart, which is unequal to the task of supporting the body with the necessary quantity of blood, fails in the parts which are farthest from its influence; the organization, in short, is *dying*, though only by inches; and the surgeon, who expects to revivify it with his knife, must first convert his gallipot into a cauldron of Medea! But suppose we take another case. A healthy young man, "in the hey-day of the blood," is wounded on the field of battle, and, after a certain lapse of time, is affected with gangrene of the limb. The gangrene appearing in the neighbourhood of the wound, sometimes, though rarely, commencing in the foot, (if the lower extremity is injured) rapidly extends, and speedily kills. For the sake of the civilians, we will take an illustration nearer home. A gentleman has the thigh transfixed, or at least wounded, by the shaft of a gig. The limb in a little while grows cold, becomes gangrenous, and dies. The same is observed after compound fractures of the leg, or in fact, as a consequence of a number of accidents. Can any one maintain, for an instant, that the gangrene of the octogenarian, or that which occurs idiophatically in a half-starved, miserable wretch, is identical, is similar to that which follows close upon a wound in a young and a healthy individual? The causes, the progress, are different; then why should the treatment be the same?

In the following extract from the excellent work of Mr. Guthrie on Gun-Shot Wounds, the question is met so fairly and fully, that we cannot resist the pleasure of transcribing it.

"A cannon-ball, striking a limb, destroys the life of the part, in a greater or less degree, according to the extent of the injury effected; if the blow be received on the middle of the leg, the bone broken, the arteries divided, or rendered incapable of carrying on the circulation, mortification takes place in the foot, because it is deprived of its usual support; it is possible, however, that this may not follow immediately, as it may not be entirely deprived of blood, which passes into it in small quantity from the parts above, connecting it with the rest of the extremity. The parts immediately above those actually struck by the ball have received a very considerable shock, and their sensibility is much impaired; and, when any action takes place in them, they will sometimes be found unequal to sustain it; and as the action attempted to be set up is inflammation, the failure of support causes it to fall into gangrene. It is, however, a failure of support, not from want of power in the constitution, exhausted by a serious struggle, but from incapability of the parts to maintain it. The extent to which this debility of parts may extend is uncertain, and the limits to the mortification must be so likewise, if left entirely to nature: it is evidently a struggle on the part of the constitution to reanimate the drooping powers of the part, which are unable to bear the assistance attempted to be afforded them. Inflammation then precedes the mortification, the limb swells, and has every appearance above the wound, as the disease advances, of humid gangrene. It began as a local disease, the part being simply unable to live, and nature having received a shock, as I conceive, entirely through the nervous system, and not by the absorbents, endeavours by means of an additional supply of blood (as she invariably does in every case of injury) to recover the parts in jeopardy, to renovate their strength. If the parts are capable of bearing this, healthy inflammation is established, and the mortification ceases. The disease is, from the moment inflammation is *established*, no longer local, the constitution is beginning to be implicated; and, if the struggle be continued, it becomes a case of mortification, dependent, according to my principles, on constitutional causes. Nature seems to suffer in the deprivation of the principle of life whenever she becomes sensible of the death of any part of the body, and in a greater proportion than would seem to be commensurate with the supply of that part in a state of health; she becomes weakened of course more by the death of a part than by its amputation; and upon a principle connected with life, which we cannot explain. When the inflammation commences, the great point for observation is whether the power of the part can or cannot maintain and carry it on to the healthy, adhesive, and ulcerative stages. If nature can accomplish this, she ought not to be interfered with; but if it appear that the part is incapable of supporting the efforts of nature, or, what is worse, that she is incapable of making them; is she to be allowed to exhaust herself in a fruitless struggle, or is assistance to be given the instant the inability of the part is seen, and just as the powers of nature are displaying themselves? I have no hesitation in saying, that the disease is yet a local one, nature is only showing what she will do if properly seconded; and that, if her efforts are directed to sound parts, capable of sustaining them, she will be able to make a sufficient and successful struggle. Amputation, then, is to be performed in sound parts, to which the usual efforts of nature will be directed; and, if they be unbroken, or only impaired by the previous injury, the result will be fortunate: but the inflammation will not be sufficiently powerful to be able to stop at the adhesive stage, union will not take place to any extent in the stump; suppuration should therefore be encouraged as a natural consequence, and no more adhesive straps should be applied than may be sufficient to keep the parts together, so as to prevent retraction. Warm fomentations and poultices should be preferred to cold applications, and the ligatures should all be cut short."

Dr. Hennen remarks, in his valuable work on Military Surgery, that to wait for the line of separation, is, in many cases, "to expose the patient to certain death;" a sentiment in which the most eminent surgeons of the day are almost universally beginning to agree.

We shall now advert to some interesting and properly authenticated cases, in which the operation has been had recourse to, prior to the appearance of a line of separation. Passing over the examples given by Baron Larrey, the first we shall notice is detailed in the work of Mr. Guthrie.

*Case* 1. A man received a blow on the back part of the leg, which stunned him, and brought him to the ground. On endeavouring to move, he found himself incapable of stirring, and the sensibility and motion of the limbs were lost. The leg gradually changed to a black colour, and when the man was carried into Brussels, the limb was apparently mortified as high as the knee; the skin was not abraded; the swelling not so great as in cases of humid gangrene; no appearance whatever of a line of separation. The inflammation appearing to be slight, amputation was performed by Mr. Campbell, at the request of Mr. Guthrie, immediately above the knee.

"On dissecting the limb, I found that a considerable extravasation of bloody fluid had taken place below the calf of the leg, and in the cavity thus formed, some ineffectual attempts at suppuration had been made. The periosteum was separated from the tibia and fibula; the popliteal artery was, on examination, found closed in the lower part of the ham by coagulable lymph, proceeding from a rupture of the internal coat of the vessel. Two inches below this, the posterior tibial and fibular arteries were completely torn across, and gave rise, in all probability, to the extravasation."

The operation succeeded, but the patient, at a subsequent period, died of dysentery. From the closure of the popliteal artery by coagulable lymph, the conversion of the extravasated blood into a sanious fluid, and the attempt which had been made at suppuration, Mr. Guthrie concludes, that the internal parts were not entirely deprived of life by the blow, but died shortly afterwards, without much effort from nature to prevent it. Here amputation was delayed until the constitution was somewhat affected, although the operation "ought to be performed as soon as the extent of the injury can be ascertained, in order that a joint may not be lost." For our own parts, we never saw a case of gangrene (traumatic), unattended with considerable disturbance of the system from the very first stage to the last. The rule, we should imagine, is to operate, as soon as the gangrene is established, care being taken, of course, to amputate above the seat of *injury*. The separation of the periosteum, from the tibia and fibula, is a curious item in the fore-going dissection, though we once saw a similar, or very nearly similar appearance.

*Case* 2. A boy, in jumping across a ditch, fell and produced compound fracture of the bones of the left leg. He was soon afterwards brought to St. George's Hospital, when the limb was placed in junks, and union of the wound, which was trifling, attempted. On the 18th, two days after admission, he complained of very great pain in the leg, and the bandages were loosened in consequence. On the 19th, some uneasiness remaining, the tails of the bandage were entirely cut away, and common simple dressing applied. At this time, he complained of some numbness of the foot, but little attention was paid to the circumstance. On the morning of the 20th, gangrene had appeared, commencing at the wound, and spreading thence downwards towards the foot, and upwards to within a little distance of the knee. The features were shrunk—pulse rapid—delirium. The gangrene continuing to spread, Mr. Brodie performed amputation as high in the thigh as could conveniently be done. The patient sustained the operation very well; whilst the state of the pulse, and anxiety of aspect, were decidedly relieved on the removal of the limb. The improvement, however, was transient and deceitful—tetanus was developed next morning, and, in little more than six and thirty hours after the performance of the operation, the boy was no more. At the wish of the friends the body was not opened, but the stump shewed no traces of gangrene whatever. The limb that was removed presented some curious appearances. All the soft parts which composed it, especially the muscular and cellular textures, were more or less disorganized. The fracture of the bones was not very severe, but the fibula was entirely separated from its periosteum, from the fracture to the epiphysis, and down to the malleolus. The separation was so complete, that, on cutting the periosteum, the bony shaft *fell out*. The same thing was noticed with the tibia, though not to so great an extent. The cartilages of the knee-joint were somewhat ulcerated; the sheath of the great sciatic nerve was studded with many little ecchymosed patches.

At first sight, this case would appear to be unfavourable to the question of amputation, though in fact, it proves little, either for or against it. After the patient's decease, it was discovered, that symptoms of tetanus had partially shewn themselves *before* the operation was performed, thus totally destroying all chance of its success. It was argued, on the other hand, that the measure was borne out in *principle*, because the mortification had not attacked the stump. The boy, however, died in thirty-six hours, too early, we should think, for gangrene to come on, particularly as, during a great part of the time, the attention of the constitution was fully taken up with another and violent disease.

In France, the opinion of the faculty is determined on the necessity of immediate amputation in cases of traumatic gangrene. M. Dupuytren, who seems to give the tone to the surgical voice in that country, invariably performs the operation.

*Case* 3. A middle aged man, in a state of intoxication, was thrown to the ground by a cart very heavily laden, the wheel passing over the thigh, and causing a comminuted fracture of the femur, *without* an external wound. Enormous tumefaction, and violent inflammation immediately followed, in which state the man was received in the Hôtel Dieu, and placed under the care of M. Breschet. The limb was *cold*, the patient delirious, and obstinately refusing to submit to amputation. This being the case, the fracture was reduced, and the thigh put up in M. Dupuytren's apparatus, but the patient could not be kept quiet, and consequently deranged the bandages and dressings. Phlyctenæ now appeared upon the leg; gangrene attacked the foot and then mounted to the leg and lower part of the thigh; the weakness was excessive; the pulse small and frequent; the belly tense and painful; the liver enlarged and felt below the ribs; the skin icteritious; the bowels very loose; the delirium so furious, that the patient required the application of a straight-waistcoat.

M. Dupuytren, disposed as he was to amputation, hesitated here, on account of the violence of the constitutional affection, and the very great engorgement of the textures of the thigh with extravasated blood. The operation was, therefore, deferred. The condition of the patient now took a favourable turn, the icteritious hue of the surface diminishing, the belly becoming less tense, and above all, the gangrene being *bounded*. The patient now was as urgent *for* an operation, as he had hitherto continued *against* it, and, notwithstanding the faint probability of success, M. Dupuytren complied. On the 25th of April, amputation was performed at the upper third of the thigh, by M. Breschet, the incision passing through muscles infiltrated and engorged, and pus flowing out from the cellular texture between them. The patient grew so weak upon the operating-table as to require to be supported with wine, and subsequently died.—*Clinique*.

Here we see a boundary actually formed, before the performance of the operation, which totally failed notwithstanding. When the man was received in the Hôtel Dieu, the limb, we are informed by the the reporter, was cold, and the constitution but little affected. This was the time for operation, if any, and we doubt whether the repugnance or refusal of a patient who was delirious, perhaps but half-recovered from his previous state of drunkenness, should absolutely deter a surgeon from its performance, provided he was fully convinced of its necessity. The question, however, is as delicate as difficult. We cannot but agree with M. Dupuytren in refusing, as he did, to advise amputation at a time when the general symptoms were so violent. The probabilities, in such a case, are greatly against us. From what we have seen, as well as from what we have read, we believe that, in traumatic gangrene, the symptoms are at times so acute, the local disease so violent and rapid in its progress, that no operation will succeed. It is the milder form of the affection which admits of the use of the knife. The symptoms, however, may be violent, without, in the first instance, the delirium, intermission of the pulse, and other characteristics of fatal prostration. In these cases, the inflammation would seem to incline to the phlegmonous, rather than the erysipelatous; the condition of the system to be inflammatory, rather than typhoid.

The following case has excited a considerable sensation in France, and was argued before the Cour Royale of Metz.

*Case* 4. A native of Villers, in the department of Moselle, received a compound fracture in the middle of the left leg, with projection of one of the extremities of bone. The medical man in attendance, reduced the fracture, and put up the limb in the ordinary way, but on the sixth day after the occurrence of the accident, Dr. Ribelotte-Lablesse was summoned to the patient. The dressings were removed, and the leg, the knees, and lower part of the thigh, were discovered to be cold, black, livid, and completely gangrenous; the pulse was small and weak; the general condition of the patient most alarming. Without amputation no hope remained; for during the time the consultation was going on, little more than an hour, the gangrene had spread two inches up the thigh. This being fully explained to the parents, and the desperate nature of any operation acknowledged, they unanimously prayed for its performance let the risk be what it might. Amputation of the thigh was accordingly performed, and, after a tedious convalescence, the patient completely recovered, and at present continues in excellent health.

It might be thought that so bold and happy an attempt, would at least have secured a trifling remuneration for his service to the operator. The ingratitude, however, on the part of the public, which Hippocrates complained of two thousand years ago, pursued Dr. Lablesse on the present occasion, and compelled him to appeal to the laws of his country, to obtain, by their means, what his patient unjustly and roguishly withheld. The cause coming on before the Cour Royale of Metz, the court directed a commission to be formed of three of the principal physicians of that city, in order to determine the merits of the case. Subsequently, M. Chaussier was directed to give an opinion on the subject, which he did in a published medico-legal consultation. This opinion, we need scarcely add, was in favour of Dr. Lablesse's operation, and somewhat to the confusion of his dastardly brethren, who stupidly and malignantly had egged on the family to resist his professional demand. The cases related in M. Chaussier's Thesis we omit; but one which is given by Dr. Buet in the 82nd number of the Clinique, deserves to be recorded. It illustrates well the frightful and fatal rapidity of traumatic gangrene, when left to itself, and affords a strong, though a *negative* proof, in favour of the operation.

*Case* 5. In the month of August, 1820, a soldier, repairing to his regiment, fell from the top of a heavy carriage, the wheel of which grazed along his leg, detaching all the soft parts composing the calf, and exposing both bones, which were not at all fractured. This was at six P. M. and the soldier was conveyed to a neighbouring cabin, where the wound was covered with powdered charcoal. Next day he was carried to the Hospital of St. Jean-de-Maurienne, in Savoy, distant about a league from the spot where the injury was received. Gangrene had already invaded the foot, leg, knee, and part of the thigh; the pulse was full, strong, and not very rapid; the skin burning hot, and tinged rather yellow; the prostration of the system considerable. The surgeon, a kind of fatalist, gave up all for lost, and merely ordered, for form's sake, some tonic and stimulant remedies internally, with fomentations of camphorated spirit and pulvis cinchonæ to the wound. At noon that day, the gangrene had spread to the crest of the ilium, many livid spots appeared on the hypogastrium, and at five in the evening, *twenty-three hours* after the accident, the patient expired.

There are few who will dispute that the above, in the first instance, was a tolerably appropriate case for operation. A very few hours decided the fate of the patient, the inflammatory state very rapidly passing, by its own excessive violence, into the typhoid. A similar, though not so acute a case, occurred some time back at St. George's, and was not considered fit for operation by the surgeon.

*Case* 6. A muscular man received a compound fracture of the left thigh, about two inches above the knee-joint; much hæmorrhage occurring at the time of the accident, and the fracture appearing to be much comminuted. The limb was placed on the double-inclined plane, cold lotions were applied, and the day after his admission, the limb being much swollen and re-action considerable, he was twice bled, to the extent of sixteen ounces, from the arm. On the morning of the third day he was seized with delirium, and the thigh had become emphysematous. *Anodynes with spiritus ætheris and camphor.* On the fourth day, mortification was developed, and the patient sank upon the sixth.

On dissection, the body, from the top to the toe, was blown up to the most extraordinary degree, with emphysema. The mortification had extended on the left side a little way up the abdomen, involving the scrotum, the penis, and nates. The femur was broken transversely an inch and a half above the knee-joint, into which the condyles had been split perpendicularly. The cancelli of the bone, the vasti, cruræus and other muscles around, as well as the cellular membrane immediately in the neighbourhood, were gorged, and injected with grumous blood; sanious matter was contained in the knee-joint, the femoral and popliteal vessels appeared to be sound; but some of the articular, or anastomotica magna arteries, or both, had been torn or divided. The cellular texture, especially that dipping down between the muscles, as well as the muscles themselves, were extremely emphysematous. Other than this, the muscles and textures of the thigh were very healthy. The viscera of the thorax and abdomen were natural, the head was not examined.

The absence of disease in the deeper-seated textures, even when the gangrene had fully run its course, seems to be in favour of an early operation. Mr. Rose, and his colleagues, however, thought otherwise, and doubtless, had sufficient reasons for their opinion. The case which we abridged in the last number of the Journal, from the clinical report of Dr. Ballingall, is, if any thing, favourable to the question at issue. Amputation was performed by Mr. Liston, and the patient survived for nearly a fortnight, dying, at last, neither from the effects of the operation nor original disease, but those depositions in the liver and the lungs which really would seem to have grown epidemic, since the publication of Mr. Rose's paper on the subject!

Previous to concluding the present article, we shall briefly allude to some other examples of successful amputation, scattered here and there in various publications. At page 155 of our 17th number, we copied from the Edinburgh Journal the case of a soldier, attacked with gangrene from the knee to the instep, after compound fracture of the tibia and fibula. Prior to the performance of the operation, the gangrene had gained the middle of the thigh; the stomach was irritable; the countenance sunk; the pulse low and weak. Amputation was performed by Mr. Macdermott, immediately below the trochanters, with success, though a good deal of sloughing occurred about the stump. The gangrene had commenced on the sixth morning after the infliction of the injury.

Dr. George Bushe, assistant-surgeon in the army, has, also, put on record two successful cases.

*Case* 7. A miserable woman, five and twenty years of age, was admitted, in the Spring of 1823, into the Richmond Hospital at Dublin, with dislocation of the foot, and rupture of the ligaments. In the course of 18 hours, gangrene appeared, and spread with activity, attended with prostration of the bodily powers. In the morning of the third day, after the occurrence of the accident, amputation was performed, by Professor Todd, below the knee, and the patient perfectly recovered.

*Case* 8. A bricklayer, at Chatham, 28 years of age, fell, on the 12th December, 1827, from a scaffold 47 feet high, and received a dislocation of the right femur upwards and backwards, a dislocation of the knee also backwards, and compound dislocation of the ankle-joint! Carried to the hospital, he was seen by Messrs. Hope and Bryant, who reduced the dislocation of the knee, dressed the wound in the foot, and put up the limb in the position of semi-flexion, after having fruitlessly endeavoured to reduce the luxation of the femur. All went on well, with care, and copious bleedings from the arm, until the third day from the occurrence of the accident, when a gangrenous spot appeared in the vicinity of the wound. In the evening, when the patient was visited by Mr. Bushe, the whole leg was covered with phlyctenæ, the thigh tense and tumid, the cellular membrane of the lower part of the abdomen in a state of emphysema. The body was cold, and covered, in parts, with a clammy sweat—the tongue quivering, the pulse weak, collapsed and rapid.

Under these circumstances, amputation of the thigh was performed just below the trochanters, a good deal of blood being lost during the operation. We shall not pursue the details of the case; suffice it to say, that the stump healed well, and the patient left the hospital completely cured.

We shall now conclude this article, certainly prolonged to a greater length than, at first, we expected it would have been. The labour of collecting these cases from so many various sources, has not been inconsiderable, and we hope is not utterly mis-spent. The result is decidedly in favour of the operation in traumatic gangrene, without waiting for the appearance of the line of separation. That the question is one of great interest no one will deny, however unimportant and useless it may seem to the mechanical and routine practitioner. The conductor of a medical journal finds, like the old man and his son in the story, that endeavouring to please all is a hopeless piece of business. Some would have only plain matters of fact, things which are constantly occurring in practice—others would consider this absolute dullness—and others again, can read or relish nothing, except gladiatorial exhibitions of personal abuse:

Hopes sapp'd, name blighted, life's life *lied* away!

We firmly believe that the latter class is daily diminishing in numbers, and the body of the profession acquiring a more wholesome literary taste. The time is passing by when science to be palatable, required to be tricked out in the meretricious attire of slang and ribald jest—it is passing, and we trust that it never will return. To go back to the subject of the present paper, we scarcely believe an apology required for dwelling on the higher parts of practice, as well as on the lower; for if either is neglected, much mischief will result. To dwell on nothing but every day, common-place topics, would destroy the scientific character of medicine, and degrade it to the level of a vulgar trade, a consummation never to be wished for.

### II. GANGRENA SENILIS.

Doctor Victor Andry has lately published a long Memoir on GANGRENE, and more especially the spontaneous, or SENILE GANGRENE, described by Pott, and other writers.

The Doctor undertakes to demonstrate that all gangrenes, under whatever form they present themselves, are referrible to one proximate cause—the cessation of the circulation in the part affected. All the occasional causes of gangrene, he maintains, act in the same manner, namely, by producing a mechanical obstruction to the course of the blood, or inflammation of the vessels themselves. This last, he avers, is the most frequent cause of gangrene, and that by interruption of the circulation. The experiments of Kaltenbrunner, and many others, prove that, in all inflammations, there is a portion of the part inflamed, in which the circulation is nearly, if not quite stagnant. If the inflammation be very violent, or if, from some other cause, the circulation be not re-established in the minute vessels, gangrene, of more or less extent, ensues.

In respect to the gangrena senilis, or Pott's gangrene, Dr. A. observes that the cessation of the circulation, in consequence of inflammation of the vessels, has not been generally acknowledged as the cause. Although the disease is certainly much more frequent among old than among young people; yet it is occasionally seen in all periods of life—and, in all cases, he maintains, it is owing to inflammation of the veins. M. Baffos lately communicated to the Royal Academy of Medicine, the case of a young woman, 20 years of age, affected with dry gangrene of the feet, attended with excruciating pains, but without any change in the colour or in the temperature of the skin. On dissection, the veins of both the lower extremities were found inflamed, and filled with a kind of substance that adhered most tenaciously to the internal surface of the vessels. M. Leveillé has reported a somewhat similar case. The spontaneous gangrene was seated in the left leg. On dissection, they found the external iliac artery and the crural vein intensely inflamed and very much thickened, being lined with a fibrinous substance which did not, however, obliterate the calibre of the vessels.

The doctrine of ossification of the arteries, as the cause of the gangrena senilis, is not always borne out by facts. Bichat has shewn that, in few individuals, after the age of 60 years, are there wanting traces of ossification of the arteries. In fact, there are many cases recorded, where the arteries of limbs were completely ossified, without any symptom of gangrene being present.

Without stopping to examine the symptoms and progress of this painful and generally fatal disease, we shall come at once to the principles of treatment—principles which flow naturally from the proximate cause here laid down.

"When then," says the author, "severe pains have preceded the appearances of gangrene, with hard and full pulse, watchfulness, &c. we may conclude that there is inflammation of the arterial or venous coats, and we ought to have recourse to blood-letting, both local and general, if the strength of the patient will permit, together with stimulating frictions and fomentations on the surface of the parts threatened with gangrene."

The following case, communicated by M. Dupuytren, from the HÔTEL DIEU, will put the good effects of depletion in a clear point of view.

*Case.* A female, aged upwards of 60 years, was received into the Hôtel Dieu, and there remained nearly a year, for the treatment of GANGRENA SENILIS, affecting the toes of the left foot. Acute and long-continued pains had preceded the gangrene, and deprived the patient, for some months, of sleep. The toes of the foot were like those of a mummy, while the neighbouring parts were of a violet colour, and emitted an insupportable smell. For the first few months of the patient's sojourn in the Hospital, opium, bark, and a variety of remedies were tried, without the least benefit. On the contrary, the disease made progress. The whole foot, soft and hard parts, were stricken with the gangrene. The state of the heart, the lungs, and the great arteries was carefully examined, but no deviation from healthy function could be discovered. M. Dupuytren now, being tired out with the various anodyne and tonic remedies usually employed, determined on opposite measures. Eight ounces of blood were taken from the arm. The pains were mitigated—and some sleep was procured. In fact, the patient experienced more relief from this bleeding, than from all the other remedies put together. This amelioration lasted nearly a fornight, when the pains began to resume their former severity. The bleeding was repeated, and with the same good effects as before. After this, phlebotomy was had recourse to, whenever the symptoms were distressing; and, by this measure, the progress of the gangrene was arrested, the mortified parts separated, and the patient was discharged cured.

Since the above period, a great many people affected with GANGRENA SENILIS have been treated in the same manner, and always with success. M. Dupuytren concludes by recommending the depletory treatment, whenever the disease is attended with severe pain, considerable tumefaction of the neighbouring parts, fulness and hardness of pulse, and flush of the face. We think there can be no doubt that this is sound advice.—JOURNAL DE PROGRÉS.

### III. URINARY BLADDER MISTAKEN FOR AN ANEURISM OF THE AORTA!! By A. LENOIR, Interne of the Hôtel Dieu.

A Man, aged about 50 years, lately died in the HÔTEL DIEU, after three months sojourn there, and his case is worthy of record, on many accounts. Five years previously, he stated that he experienced suddenly a rather severe pain in the left lumbar region, which, however, did not prevent him from following his avocation of cook, in one of the Parisian Colleges. Afterwards, the pain became more severe, and extended to the lower extremity of that side. He applied to various hospitals and public charities, during the next two years, and the disease was always pronounced to be SCIATICA, and treated (unsuccessfully) as such. Leeches and blisters innumerable had been applied, without effect, and at length he took up his final residence in the HÔTEL DIEU, in March, 1828. Then, as during the preceding five years, he complained only of excruciating pain, stretching from the left lumbar region, down along the lower extremity of that side. After an examination into the history and symptoms of the case, the disease was once more pronounced to be SCIATICA, and treated accordingly. All the organs appeared to be performing their functions regularly, except the bowels, which were so constipated that the patient had only one evacuation every twelve or fifteen days. This circumstance induced the medical attendants to institute a more rigorous examination, and the result was the discovery of an oval tumour just below the umbilicus, about the size of a man's fist. It presented distinct pulsations synchronous with the arteries. The diagnosis was now altered from SCIATICA to aneurism of the lower portion of the aorta, or of the left primitive iliac. This explained (so they thought) the pain in the course of the sciatic nerve, and the œdema of the limb. They did not employ the stethoscope in this examination, and the infiltration was too great to permit the crural artery to be felt, so as to ascertain the state of the circulation below the aneurismal tumour. The patient was too much reduced to admit of depletion, and an analeptic regimen merely, with opiates, were all that could be employed. He at length sunk exhausted by the excruciating and incessant pain.

*Dissection.* Before the abdomen was opened, they again examined by external manipulation, and again the aneurismal tumour was felt as distinctly as in the living body—with the exception of pulsation. The parietes were then incised, and, to the astonishment of all present, the aneurism was converted into a contracted bladder, whose coats were thickened, and whose situation was changed in consequence of a cancerous tumour in the pelvis, which seems to have sprung from the left ilium, displaced and jammed the rectum, and nearly filled the cavity of the pelvis. The bladder, in fact, was pushed up out of the pelvis, and rested on the left iliac artery, from which it derived its pulsatory movements. The mass of disorganization, involving bone, muscle, nerve, blood-vessels, &c. could not be unravelled; but when the ilium was cleared by masceration, it was found to be more than two inches in thickness, and its texture resembled that of a sponge with very fine areolæ.

We think there is little doubt that the stethoscope would have proved the non-aneurismal nature of the tumour, and saved the ridiculous diagnosis that was given. It is true, the error did no harm in the above case; but had the patient lived on this side of the channel, the celebrated operation of ligature "ultra tumorem," would certainly have been performed. After such an operation, it is probable that the aneurism would not enlarge, and the triumph of surgery would be complete. If death and dissection revealed any unpleasant phenomena, the whole would, of course, be candidly recorded in the pages of some periodical.

### CLINICAL REVIEW, No. II.

### XXXVIII.

### HOTEL DIEU.

MEMOIR ON PHLEGMONOUS TUMOURS, OCCUPYING THE RIGHT ILIAC FOSSA. By P. MENIERE, M.D. Interne de l'Hôtel Dieu, &c.

[Practice of M. Dupuytren.]

This dangerous complaint has lately attracted much attention on the Continent, and M. Dupuytren gives, each season, a lecture on the subject. The disease is by no means unfrequent, and we need hardly say, that it is very generally overlooked or misunderstood, till too late.

It is to be remembered, that in the right iliac fossa lies the cæcum, two-thirds of whose circumference is covered by the peritoneum—the posterior part being in immediate contact (by means of cellular tissue) with the iliacus internus muscle. The abrupt termination of the ileum—the form and position of the ileo-cæcal valve (valve of the colon)—the size of the cæcum, and structure of its parietes, all contribute to the *remora* of the fæces in this place, and partly explain the changes which are effected in the colour, consistence, smell, &c. of the fæcal remains, at this particular locality in the intestinal canal. In order to pass into the colon, the fæces must overcome their own gravity in the course of a vertical ascent, a circumstance which requires a considerable degree of propulsive force, and, consequently, a *fixity* in that part of the canal. The adhesion of the cæcum to the iliacus internus explains this circumstance. It is hardly necessary to observe, that this expulsive force acts on the valve of the colon, which shuts in consequence, and prevents the return of fæcal matters into the small intestine. The peculiar offices performed at this portion of the intestinal canal, render the part liable to morbid affections, which have not been sufficiently studied by the pathologist and physician.

It is well known that inflammation of the mucous membrane which lines a secretory or excretory tube, is readily propagated to the cellular tissue in the neighbourhood, and thence to other contiguous parts. The lachrymal sac—the parotid duct—the œsophagus—the orifices of the stomach—the rectum, and the urethra, have furnished innumerable examples of this fact. The same applies, our author observes, to the cæcum, as will be shewn by authentic cases in the course of this paper.

Ileo-cæcal inflammations, acute and chronic, are extremely common—but, while this is acknowledged by almost all pathologists, there is much diversity of opinion respecting the importance of the malady, the causes which produce it—and the symptoms which attend it. There are some physicians who attribute the cause of this inflammation to the constant contact of fæcal remains—others to some morbid condition of the fæces, by which they acquire an irritating quality. How-

---

ever this may be, we may safely lay down, in principio, *first*, that inflammation of the mucous membrane of the cæcum propagates itself occasionally to the contiguous cellular tissues—*secondly*, that this last phlogosis, once developed, does not necessarily follow the steps of that which originally produced it—*thirdly*, that it pursues a course, dependent on the texture of the parts affected, and on accidental circumstances. But we shall now proceed to facts, from which the reader will be able to draw his own conclusions.

*Case* 1. Charles Billet, aged 16 years, of feeble health, became affected with indigestion, after imprudent diet, in the beginning of August. From this time till the 20th October he was afflicted with alternate constipation and diarrhœa, attended with colicky pains. On the 21st, these pains became much increased, and the bowels obstinately confined. There was a fixed pain in the right iliac region. On the 27th, the constipation having persisted, a swelling was observed in the above-mentioned place. Lavements, fomentations, &c. were employed at home, but without benefit, and on the 31st he entered the HÔTEL DIEU. He had now acute fever, hot skin, pale furred tongue, abdomen painless on pressure, except in the right iliac region, where there was a tumour the size of a man's fist. It was hard, immoveable, excessively painful on pressure. Bowels obstinately confined—no discharge even of flatus per anum. Forty leeches were applied to the part, succeeded by cataplasms. Lavements were thrown up. 1*st of November*. The leech-bites bled copiously—the tumour is much less painful. Twenty more leeches were applied, and the lavements continued. Next day there was a copious discharge of solid fæces—the swelling is scarcely tender on pressure—the fever greatly diminished. By a perseverance in laxatives the bowels were kept freely open—the swelling diminished—and the patient was discharged on the 12th of November.

We have no doubt that there was inflammation about the cæcum in this case, of which we have seen many parallel instances; but we apprehend there was an accumulation of fæces in this part, producing the principal bulk of the swelling. The author of the memoir before us does not coincide in this opinion. He thinks the tumour was the result of inflammation in the cellular tissues adjacent to the cæcum. The four following cases are given in support of his opinion.

*Case* 2. A young man of 18, apparently scrofulous, and who had been recently taking bitters and tonics of all kinds, became affected with colicky pains of a wandering kind, which lasted eight days, when a fixed pain was complained of in the right iliac fossa. This lasted six days more, during which there was obstinate constipation. A swelling now became developed in the seat of the pain, accompanied by fever, anorexia, &c. The bowels were opened at this period. The application of five batches of leeches, with fomentations, poultices, warm baths, and rigid regimen, completed the cure in the course of a month.

*Case* 3. A man, aged 30 years, who had been often in the Hôpital St. Louis for lepra vulgaris, and who had taken a great deal of acrid purgative medicine that always occasioned colicky pains and diarrhœa, became affected with fixed pain in the region of the cæcum, accompanied by a tumour in that part, the size of an egg. Diarrhœa continued obstinate, and the patient thought he felt the passage of flatus and fæcal matters past the tumour. Repeated applications of leeches, fomentations, and poultices, gave much relief; but, at the date of report, there existed still a tumour in the part, though the general health was completely restored.

*Case* 4. A ship-painter, aged 20 years, who had been often affected with colicky pains and constipation, was treated, in one of these attacks, by repeated exhibitions of strong emetics and cathartics. The symptoms, instead of being mitigated, were exasperated, and the pain became concentrated in the right iliac fossa, accompanied by a circumscribed and very painful tumour. This had continued nearly ten weeks, when active treatment was employed, consisting of repeated leechings, fomentations, poultices, and lavements. In 20 days of this treatment, the cure was effected.

*Case* 5. A robust stone-mason, aged 22 years, had a smart diarrhœa, after which the bowels became constipated, and he had pain in the right iliac fossa for six days. A tumour then appeared, accompanied by painful pulsations there. Three applications of leeches—with fomentations, poultices, and purgative lavements dissipated the pain, but the tumour went off very slowly.

*Case* 6. Louis Perrin, aged 29 years, of small stature and weak constitution, and who had twice been affected with colica pictonum, experienced a smart diarrhœa in the month of June, 1825, and this lasted six weeks. On the sixth of August he felt a violent pain in the region of the cæcum; and in eight days afterwards, a tumour became developed there. He entered the Hôtel Dieu on the 8th of the same month. No treatment had been employed. The tumour was now the size of a goose egg, very painful, round, and hard. Thirty leeches —fomentations—cataplasm—purgative lavements. Some solid motions were procured by the enemata, and the patient felt relieved. In the following days, the tumour lessened in size, became softer, and some fluctuation was perceptible. Two other applications of leeches. *2d September*. The patient committed some imprudences in diet, and was seized with a strong indigestion. During some efforts to vomit, he felt, all at once, a very acute pain in the centre of the swelling. Next day a considerable quantity of pus was found mixed with the stools. On the 5th and 6th of the same month, more pus came away, but in smaller quantities, and the tumour had greatly diminished. The patient soon afterwards insisted on his discharge from the hospital, though the swelling had not entirely disappeared.

We agree with the author that here there was inflammation and suppuration of the cellular membrane beneath the cæcum, and that the pus burst into the cavity of the intestine.

Two other similar cases are related from the memoir of Dr. Dance, (which we reviewed in a former number of this Journal,) and where the matter found its way into the colon, with ultimate recovery. In some comments which our author makes on these cases, he argues, and with much plausibility, that the arrest or impaction of stercoraceous matters in the cæcum, is the consequence rather than the cause of the cæcal inflammation. Granting that it is a consequence, in the first instance, there can be no doubt that it proves a cause of the *continuance* of the inflammation, by the mechanical irritation which it must necessarily keep up.

A case is next taken from Dr. Dance's memoir, where general peritoneal inflammation supervened on cæcal inflammation and suppuration. This, of course, is properly looked upon as an epiphenomenon.

*Case* 7. Louis Rouche, 24 years of age, was subject, for three or four months after arriving in Paris, to diarrhœa and colick. In the beginning of September, the right iliac fossa became painful. No treatment was employed. On the 12th of the same month, he entered the Hôtel Dieu. He now had fever, tongue coated yellow, painful tumour in the region of the cæcum. Twenty-five leeches to the part. These were repeated on the 13th and 14th of the same month. There was considerable mitigation of the symptoms. On the 15th, there were violent colicky pains—numerous stools—great tenderness on pressure—ardent fever—constant jactitation—apparent extension of the tumour—venesection—40 leeches. Next day, 30 more leeches were applied; but the symptoms increased in violence, and he died on the 19th of the same month.

*Dissection*. General peritonitis was found to obtain, with considerable effusion of yellowish fluid into the cavity of the abdomen, and adhesions of the intestines. The mucous membrane of the cæcum was inflamed, but not ulcerated. The cellular tissues in the neighbourhood of the cæcum were infiltrated with purulent matter, and this infiltration extended to the kidney of that side, and down into the pelvis, even to the rectum and bladder.

We need not accumulate more cases, as they would only be repetitions of the same picture. The following *resumé* of our author deserves a record in this place.

1mo. Age and sex seem to have some influence in predisposing to this malady. Of sixteen cases which the author noted, eleven were beyond the age of 30 years. Fifteen out of the sixteen were males. The malady was more frequently seen in the Summer and Autumn than at any other period of the year.

2ndo. *Occasional Causes.* These are numerous. Professions are very influential in the production of the malady. Thus painters, colour-grinders, and men who work in the manufacture of copper utensils, have been observed to be more subject to this inflammation than others. People also who lead a sedentary but anxious life, are more usually affected with disorders of the digestive function, and with the ileo-cæcal inflammation, than those who spend their time in a different manner. Bad food, and indigestible substances are, of course, the most powerful occasional causes—in short, whatever tends to produce irritation, or excite inflammation in the mucous membrane of the intestines, tends also to induce ileo-cæcal disease.

3tio. The precursory symptoms are alternate diarrhœa and constipation; vague colicky pains tending to concentrate themselves towards the iliac fossa, increased sensibility on pressure of the part. The duration of these precursory symptoms is very various—extending sometimes to six weeks or two months—sometimes being only a few days before the manifestation of swelling in the part. There is, however, little or no dependence to be placed in the foregoing symptoms, because they frequently take place without any tumour following.

4to. The symptoms *proper* to the malady are, *fixed pain* in the ileo-cæcal fossa—and tumefaction at this point. All other symptoms are accessory and equivocal, such as the fever, the anorexia, the irregularity of the bowels, &c.

5to. *Prognosis.* This, in general, is not unfavourable. Of the sixteen cases which came within our author's own observations, only one proved fatal. When the pain and swelling cede to the proper means, when the bowels become freely evacuated, when the fever recedes, we may calculate on recovery. When, on the other hand, the symptoms do not give way, when the tumour enlarges, and fluctuation is felt, we may hope and expect that there will be a purulent discharge *per anum*—and even then we may look for a favourable issue. But when symptoms of general peritonitis supervene, we may expect that the matter is not taking the safe course into the colon, and that death will be the consequence.

6to. Resolution is the most common, and the most desirable termination of these phlogoses. In eleven cases out of sixteen, this was the mode of termination. In M. Dupuytren's experience, however, the suppurative issue was more common than that by resolution.

7mo. THE TREATMENT. This will readily suggest itself when the nature of the malady is detected. This is the grand point—for we are persuaded that many cases of the disease are entirely overlooked by routine practitioners, or confounded with affections of the liver or bowels, for which drastic purgatives are given that aggravate the disease. The premonitory symptoms to which we have alluded, although not unequivocal evidences of the approaching disease, ought nevertheless to put the practitioner on his guard; for by attending to the state of the bowels at this time, and by the early application of leeches, when local pain is complained of, the future disease might be prevented in many cases. When there is any actual swelling, hardness, and pain on pressure, no time should be lost in leeching, fomenting, starving, and gentle purgation—especially by emollient lavements. The great error which medical men commit is the non-examination of patients with their clothes off. It is quite impossible to form any thing like an accurate estimate of the state of parts, in the abdomen especially, without this kind of investigation. A pain is complained of in the right side of the abdomen, accompanied by some symptoms which are vaguely denominated *bilious.* The disease or disorder is hastily set down as a "liver-complaint," when an accurate examination, in nine cases out of ten, would shew the disorder to be in the colon, or in some part of the intestinal canal.

P.S. The Editor has seen several cases of this complaint—and has recently attended one very well-marked instance with Mr. Fincham, an intelligent and experienced surgeon, residing in Spring Gardens. The patient was a young gentleman, who had gone on a boating expedition to Richmond, and there indulged freely in various kinds of indigestible ingesta. The bowels were confined for several days, and then came on pain and swelling in the region of the cæcum, with vomiting, furred tongue, and the various symptoms already described. The tumour, at one time, was the size of the head of a fœtus, and the young gentleman was in very great danger for several days. It required numerous leeches—calomel and opium—enemata and fomentations, with very rigorous antiphlogistic treatment, to rescue this patient from death.

---

